# Cultural Adaptations to Recruitment Strategies and Community-Based Interventions for Dementia Caregivers

**DOI:** 10.1093/geroni/igab046.1867

**Published:** 2021-12-17

**Authors:** Lauren Parker, Katherine Marx, Maria Aranda

## Abstract

Nearly 30 years after the 1993 National Institute of Health Revitalization Act, minority groups’ low participation in research remains (which required the inclusion of women and racial/ethnic minority groups into government-funded clinical trials). This is particularly the case for participation in research on Alzheimer’s Disease and related dementias (ADRD). Deeply rooted historical race-based mistreatment in research and in the health care system at large persist as barriers to low-participation of minorities (i.e. Black/African American, Hispanic/Latino) and immigrants in research studies, who remain at disparate risk for adverse ADRD health outcomes and expedited mortality. The use of culturally adapted approaches in recruitment strategies and community-based interventions might be helpful to encourage the participation of underrepresented groups into research. As such, this presentation highlights three studies that seek to use cultural adaptation to inform recruitment strategies and community-based interventions. First, Dr. Parker will present how tenets from Critical Race Theory can be used to inform culturally-adapted recruitment strategies of Black/African American caregivers into community-based research by drawing upon two ongoing studies: a randomized trial providing caregiver support through Adult Day Services (ADS) and the evaluation of impact of ADS on stress levels of Black/African American using biomarker measures. Next, Ms. Johnson will present results on cultural adaptions to the ADS-Plus Program for Spanish-speaking populations. Finally, Dr. Nkimbeng will present on the process of culturally-tailoring dementia education for African immigrants in Minnesota. Findings from this presentation identify opportunities for researchers to use cultural adaptations to encourage participation of underrepresented populations into ADRD research.

